# Self‐help cognitive behavioral therapy for gaze anxiety in young adults: Protocol of a 3‐arms, multicenter, randomized controlled trial

**DOI:** 10.1002/pcn5.70236

**Published:** 2025-11-04

**Authors:** Kazuki Matsumoto, Sayo Hamatani, Yoshifumi Mizuno, Akiko Maeno, Makiko Kasai, Katsunori Watanabe, Rio Kamashita, Naomi Sunami, Masatoshi Ikeda, Shinobu Nagata, Masahiko Inoue, Yasuhiro Kimura, Mizue Yokoo, Yoshihiko Kunisato, Yasuaki Akasaki, Masayuki Nakamura

**Affiliations:** ^1^ Division of Clinical Psychology, Kagoshima University Hospital, Research and Education Assembly Medical and Dental Sciences Area Kagoshima University Kagoshima Japan; ^2^ Research Center for Child Mental Development University of Fukui Fukui Japan; ^3^ Department of Clinical Psychology, Faculty of Human Relations Shigakukan University Kagoshima Japan; ^4^ Clinical Psychology Course Naruto University of Education Tokushima Japan; ^5^ Department of Psychology Jin‐ai University Fukui Japan; ^6^ Division of Occupational Therapy, Department of Rehabilitation Hiroshima Cosmopolitan University Hiroshima Japan; ^7^ Department of Psychology, Faculty of Liberal Arts Teikyo University Tokyo Japan; ^8^ Clinical Psychology Course, Department of Psychology Shujitsu University Okayama Japan; ^9^ Department of Clinical Psychology Faculty of Medical Tottori University Tottori Japan; ^10^ Department of Welfare Psychology, Faculty of Welfare Fukushima Gakuin University Fukushima Japan; ^11^ Psychological Course Tokyo Rissho Junior College, Faculty of Modern Communication Tokyo Japan; ^12^ Department of Psychology Senshu University Kawasaki Kanagawa Japan; ^13^ School of Health Sciences, Faculty of Medicine Kagoshima University Kagoshima Japan; ^14^ Department of Psychiatry Kagoshima University Graduate School of Medical and Dental Sciences Kagoshima Japan

**Keywords:** cognitive behavioral therapy, gaze anxiety, scopophobia, self‐help, social anxiety disorder

## Abstract

**Background:**

Gaze anxiety (scopophobia) is a frequently observed and reported symptom that is closely associated with social anxiety disorder (SAD). Considering the efficacy of cognitive behavioral therapy (CBT) for social anxiety, it may also be beneficial for individuals with gaze anxiety. Self‐help CBT can offer a means of early intervention; however, its effectiveness remains unclear.

**Methods:**

This protocol describes the design of a clinical trial aimed at evaluating the efficacy of two self‐help CBT interventions for gaze anxiety. This multicenter randomized controlled trial is planned across 12 institutions in Japan. The participants will be young adults aged 18–30 years who report anxiety and avoidance related to gazes. The enrolled participants will be randomly assigned to either a self‐help book CBT group, a web‐based CBT group, or a control group. The severity of gaze anxiety, as the primary outcome, will be assessed using online questionnaires at baseline, 3 months post‐intervention, and the 6‐month follow‐up, with effectiveness evaluated through statistical analysis using mixed‐effects models.

**Results:**

The clinical trial will be performed from April 2025 to March 2026, and the results of this study are expected to be available by mid‐2026. The results of this trial will provide insights into the effectiveness of self‐help CBT for gaze anxiety (scopophobia).

**Conclusions:**

This trial will examine whether self‐help CBT improves access and whether it is effective for adolescents and young adults with social anxiety, especially gaze anxiety.

## INTRODUCTION

Social anxiety disorder (SAD) is a mental disorder characterized by excessive fear of scrutiny and potential embarrassment in social situations, along with anxiety about negative evaluation by others.^1^ SAD commonly onsets during adolescence and has a lifetime prevalence of approximately 4.0%.^2^ Adults and children with SAD tend to avoid eye contact,^3^, ^4^ and the tendency seems to appear in adolescence due to excessive gaze anxiety.^5^ Being looked at often signals the start of interaction in social situations. If gaze anxiety is severe and leads to the avoidance of others, habituation to gaze anxiety may not occur. This can maintain or even worsen gaze and social anxiety. Therefore, early intervention for people with gaze anxiety before it interferes with their daily lives is important.

Individual cognitive behavioral therapy (CBT) for SAD is significantly effective and has been recommended as a treatment option in treatment guidelines.^6^, ^7^, ^8^, ^9^ However, it is unclear how to effectively deliver this promising treatment for preventive interventions. The reason for this is that individual CBT generally has high time and monetary costs. Self‐help CBT is potentially feasible for reducing gaze anxiety, similar to its effectiveness in treating SAD. Self‐help CBT, which people can perform at home, eliminates the need to miss school or university and has lower implementation costs because there is no therapist facilitating individual sessions. For social anxiety in adolescents and young adults, self‐help CBT on websites has been shown to have an effect; however, it is unclear whether it helps with gaze anxiety.^10^ Self‐help CBT via books without therapist guidance has been shown to improve SAD in two previous randomized controlled trials (RCTs).^11^, ^12^ However, there is limited evidence from RCTs in Asia. Furthermore, the efficacy of self‐help CBT for treating gaze anxiety remains unclear.

A clinical trial was designed to examine the efficacy of a self‐help CBT using a book, developed with the aim of alleviating gaze anxiety, and an internet‐based CBT (ICBT) program for social anxiety. This manuscript details the protocol for this clinical trial.

## METHODS AND ANALYSIS

### Trial design

This study protocol is a multicenter RCT being conducted at 12 research institutions in Japan. Eligible participants will be randomly assigned in a 1:1:1 ratio to one of three groups: self‐help CBT book group, ICBT group, or control group (no‐treatment control).

### Participants

Participants will be recruited from the following universities: Kagoshima University, University of Fukui, Shigakukan University, Naruto University of Education, Jin‐ai University, Hiroshima Cosmopolitan University, Teikyo University, Shujitsu University, Tottori University, Fukushima Gakuin University, Tokyo Rissho Junior College, and Senshu University. Additionally, participant enrollment will be solicited through the research project website. The predetermined inclusion and exclusion criteria outline the specific requirements that potential participants must meet for enrollment in the trial.

Inclusion criteria:

1. A total score of 10 or higher on a measure of gaze anxiety symptoms using the Gaze Anxiety Rating Scale (GARS).^3^
2. Enrollment as a university and graduate student aged between 18 and 30 years.
3. Capacity to send and receive electronic mail.
4. No planned transfer or withdrawal from their academic institution during the study period.

Exclusion criteria:

1. Presence of mental disorders, including but not limited to major depressive disorder and SAD.
2. A history of suicidal ideation or attempts.
3. Presence of a progressive medical illness (e.g., cancer).
4. Pregnancy or the immediate postpartum period.
5. Any condition or behavior deemed by the researchers to render the individual unsuitable for participation in this study (e.g., antisocial behavior).

For participants identified as being at risk for mental health conditions, the researchers from each university will inform the principal investigator (K.M.), who has been the study secretariat, to assess their eligibility by using the Mini‐International Neuropsychiatric Interview (MINI).^13^, ^14^

### Intervention

#### Self‐help by using a book group

Participants allocated to the self‐guided book intervention will be instructed to read one chapter (approximately 30–50 pages) per week from the self‐help book on gaze anxiety in young adults, “Escaping the Fear and Anxiety of Others' Gaze.”^15^ This self‐help book was authored by the first author (K.M.) and published in October 2024. Table 1 presents the contents of the chapters of the self‐help book. This book has 193 pages long with wide line spacing, and we estimate it takes approximately 150 min to read. Each Monday at noon, participants in this group will automatically receive an announcement detailing the chapters to read for the week and words of encouragement.

**Table 1:** Time to read on the book and its contents.

Week	Title each chapter	Introduced techniques	Page
１	Why do I feel anxious when people look at me?	Psychoeducation	31
２	Understanding the roots of your gaze anxiety	Cognitive behavioral model	21
３	Steps to overcoming your gaze anxiety	Examining safety behaviors Video feedback Attention shift training Behavioral experiments Social/opinion surveys Ceasing rumination and pre‐ and post‐event processing Processing social trauma in image description	81
４	Let's conquer social situations one by one: Case studies	Anxiety about being seen trembling Fear of making public mistakes and being observed Fear of blushing when looked at Fear of saying the wrong thing and being judged	56
５	As your “gaze anxiety” improves	Relapse prevention	2

#### Self‐help in ICBT group

Participants allocated to this intervention group will be instructed to engage using their own devices in an ICBT program designed to ameliorate social anxiety in adolescents and young adults. The completion of one module per week will be recommended, and automated email reminders encouraging program engagement will be sent to participants every Monday morning. The ICBT program was developed by the first author (K.M.) on the e‐learning platform learningBOX (learningBOX Inc., Tatsuno, Japan). The ICBT program is based on the model proposed by Clark and Wells.^16^ The program comprises 10 training sessions focusing on CBT components that are efficacious for SAD. Each module is expected to take about 15 min to complete, and the entire content can be finished in about 150 min. Table 2 outlines the treatment modules of the ICBT program.

**Table 2:** Treatment module of the internet‐based cognitive behavioral therapy (ICBT) program.

Week	Module
1	Psychoeducation and case‐formulation using the cognitive behavioral model
2	Examining safety behaviors and self‐focused attention
3	Video feedback to correct negative self‐image
4	Attention shift training
5	Behavioral experiments
6	Opinion survey
7	Ceasing rumination and pre‐ and post‐event processing
8	Processing social trauma in image description
9	Schema work
10	Relapse prevention

#### Control group

The control group will receive no active intervention during the study period; however, the self‐help book will be sent via mail to the participants upon study completion. While participants in the control group will be permitted to seek usual care, such as counseling, they will be asked to refrain from accessing information specifically related to CBT.

### Outcome

All outcome measures will be structured as web‐based questionnaires and sent to the participants' email addresses at baseline, post‐intervention (5 weeks follow‐up), 3 months follow‐up, and 6 months follow‐up. To promote participant retention and follow‐up completion, monthly email reminders will be sent from the start of the intervention until the end of the follow‐up period. Any deviations from the trial intervention will be recorded during follow‐up assessments.

The primary outcome will be the GARS used to measure the severity of clinical symptoms and rate anxiety/fear and avoidance of gaze. The GARS comprises 17 items regarding social situations involving focused gaze.^3^ For each item, participants will rate the degree of anxiety/fear and the frequency of avoidance on a scale from 0 (“no anxiety” or “no avoidance”) to 3 (“a lot of anxiety” or “avoid a lot”). The total GARS score ranges from 0 to 102, with higher scores indicating a greater severity of gaze anxiety. The validity and reliability of the GARS have been demonstrated in the original English and German versions.^3^, ^17^, ^18^ The Japanese version of the Gaze Anxiety Rating Scale (GARS‐j) was developed through translation into Japanese by the first author (K.M.) and back‐translation by the second author (S.H.), with the resulting text being reviewed by the original scale developers (please refer to the GARS‐j in the Supporting Information).^19^ Furthermore, the validity of the GARS‐j will be examined using baseline responses from this trial. Table 3 shows secondary outcomes.

**Table 3:** Secondary outcomes.

Outcome	Description
LSAS‐j	The LSAS‐j includes 24 social situations related to “fear or anxiety” and “avoidance,” which participants rank on a 4‐point scale (0 = never, 4 = severe), has a total score range of 0–144.^20^ The Japanese version of the LSAS has demonstrated reliability and validity and is widely used in Japanese clinical and research settings.^21^ Therapeutic response will be defined as a ≥28% decrease in the LSAS total score.^22^ Remission will be defined as an LSAS total score of <35,^23^ consistent with a previous RCT.^10^ Conversely, worsening will be defined as a ≥28% increase in LSAS total score to assess the risk of exacerbating social anxiety.
SPIN	The SPIN is a self‐rating scale that measures 3 characteristic aspects of SAD: fear, avoidance, and physiological arousal.^24^ In the Japanese version of the SPIN, 17 questions are answered on a 5‐point scale, with 0 = not at all applicable and 4 = extremely applicable.^25^
PHQ‐9	Depressive symptoms will be assessed using the Japanese version of the PHQ‐9.^26^ The initial nine items of the PHQ‐9 inquire about the frequency with which symptoms of major depressive disorder have occurred over the past 2 weeks. For each item, participants select a response from 0 (not at all) to 3 (nearly every day), yielding a total score ranging from 0 to 27, with higher scores indicating a greater severity of depressive symptoms.^27^
GAD‐7	Generalized anxiety will be assessed using the Japanese version of the GAD‐7.^26^ The initial seven items of the GAD‐7 inquire about the frequency with which symptoms of generalized anxiety disorder have occurred over the past 2 weeks. For each item, participants select a response from 0 (not at all) to 3 (nearly every day), yielding a total score ranging from 0 to 21, with higher scores indicating a greater severity of generalized anxiety symptoms.^28^

*Note*: We will also calculate recovery rates based on the Improved Access to Psychological Therapies program criteria and improvement (recovery) rates defined as achieving total scores of SPIN < 19 and PHQ‐9 < 10 simultaneously.^29^ This definition has been used as a benchmark for improvement from SAD.

Abbreviations: GAD‐7, Generalized Anxiety Disorder‐7; LSAS‐j, Japanese version of the Liebowitz Social Anxiety Scale; PHQ‐9, Patient Health Questionnaire‐9; SAD, social anxiety disorder; SPIN, Social Phobia Inventory.

### Sample size

**Figure 1:**
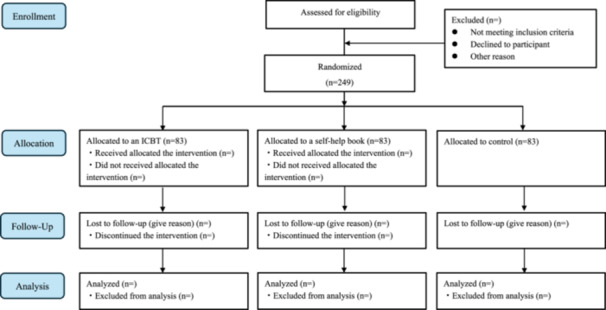
CONSORT flow diagram showing allocation in the three groups. ICBT, internet‐based cognitive behavioral therapy.

The sample size for this study was set at 249 (Figure 1), considering the following G*Power estimated values and a 20% attrition rate (version 3.1.9.7; The G*Power Team).^30^, ^31^ We set the parameters for the *F* tests—anova: fixed effects, omnibus, one‐way, with an effect size *F* = 0.25 (medium), an *α* error probability of 0.05, and a power (1 − *β* error probability) of 0.9 for three groups. These inputs yielded a noncentrality parameter of 12.94, a critical *F* of 3.04, an original total sample size of 207, and an actual power of 0.902.

### Randomization

Eligible participants will be randomly assigned to each group at a ratio of 1:1:1, with assignments made using a block randomization method with R' “randomizr” package,^32^ ensuring a balance in baseline Japanese version of the Liebowitz Social Anxiety Scale (LSAS‐j) total scores of <50 or ≥50, sex (male or female), and facilities as adjustment factors. Allocation by using the randomizer will be conducted by the second author (S.H.). The randomization sequence will be concealed from researchers at each university, and the statistician will remain blinded to participants' group assignments.

### Blinding

This trial will not be blinded.

### Adverse events

We will instruct all participants at each university to immediately report any adverse events that occur during this trial period. If additional support is needed, participants will be referred to campus counseling services or encouraged to visit local clinics affiliated with their universities. Even if no adverse events are reported, participants scoring 10 or above on the Patient Health Questionnaire‐9 (PHQ‐9) total score during the follow‐up period will assess their mental health problem by using the MINI.^13^, ^26^ Based on the results of this assessment, participants may be recommended to see a medical doctor.

### Adherence

We will assess adherence to the two interventions using two methods. In the book group, participants will be asked to report the chapters of the self‐help book read and cognitive‐behavioral techniques practiced in daily life at the initial outcome assessment. For the web‐based CBT intervention, the e‐learning system will record completed treatment modules automatically, and participants will also report cognitive‐behavioral techniques practiced in daily life at the initial outcome assessment. All participants, including the control group, will be asked to report any unintended treatments or care received during the study period.

### Statistical analysis plan

Statistical analyses will be performed by the second author (S.H.) using IBM spss Statistics (IBM Corp., Armonk, NY, USA) and will adhere to the intention‐to‐treat principle. The analyst was not involved in creating the two intervention programs. The analyst will be concealed to group allocation when accessing the data. This means she will not know which specific intervention a group received or if each participant was in the control condition.

Descriptive statistics will be used to summarize demographic data (including participants' age and sex) and the severity of clinical symptoms on each scale. Group differences will be compared to determine statistical significance. As a primary analysis, mixed‐effects models will examine significant differences in the mean change in total scores on each symptom rating scale between the groups from baseline to Week 5 (1 month), Week 13 (3 months), and Week 26 (6 months) post‐intervention. If an overall significant difference is found, multiple comparisons will be conducted. For multiple comparisons, we will implement Dunnett's test to control for inflated Type I error rates.^33^ Effect sizes will be calculated between and within the groups. The significance level for hypothesis testing will be set at a two‐tailed *α* of 0.05, and 95% two‐sided confidence intervals will be calculated.

For the binary variables of treatment response, remission, and worsening, the odds and risk ratios between the groups will be analyzed using Fisher's exact test. To further evaluate the risk of worsening social anxiety, as measured by the LSAS, we will calculate the risk ratio, relative risk reduction, absolute risk reduction, and number needed to treat.

We plan to conduct a sensitivity analysis excluding participants with insufficient engagement in the self‐help intervention and those who provided implausible response patterns—specifically, patterns like straight‐lining, contradictory responses, or inconsistent responses between the GARS‐j and the LSAS‐j. Participants will be defined as having sufficient engagement if those in the self‐help book group have completed at least two chapters, and those in the ICBT group have reached the “Behavioral Experiments” treatment module. The sensitivity analysis, similar to the primary analysis, will be performed after imputing missing values using R's “mice” package.^34^

### Ethics and dissemination

The trial protocol was approved by the Institutional Review Board of Kagoshima University Hospital (Approval Number: 240229). This clinical trial was registered at the University Hospital Medical Information Network Center (UMIN), and an overview of the research protocol is publicly available (UMIN000057484). This study's intervention program addresses cognitions and behavioral habits related to gaze and social situations that participants may find distressing, while also promoting the practice of social skills. During the research explanation, participants will be informed that they can contact researchers at any time and that they have the right to withdraw consent at any point.

## RESULTS

The clinical trial will be conducted from April 1, 2025, to March 31, 2026. The case registration period will be scheduled for 2 months, from April 1 to May 31, 2025. The results of this trial, in conjunction with the execution of the predesigned statistical analyses plan, will provide novel knowledge into the effectiveness of self‐help CBT for gaze anxiety (scopophobia).

## DISCUSSION

The limitations of this study include the inability to determine the true effects of the two self‐help CBT programs because the control is not a placebo (sham treatment). Because recruitment for this study will take place at universities affiliated with researchers specializing in CBT, many potential participants will have a clinical psychology background and may be able to discern the researchers' intentions if an attention control or psychoeducation condition is used as a placebo. This is because the lectures will have explained that CBT is an effective treatment for SAD, and they can find out through an internet search that CBT for SAD is covered by insurance in Japan. On the other hand, a no‐treatment control group could lead participants to seek treatment outside the study, including face‐to‐face CBT, during the observation period. Furthermore, disappointed participants might withdraw from the study, which could increase the risk of bias. Given these practical constraints, we believe it is more appropriate for the control group to be guaranteed an intervention (self‐book CBT) after the study.

Another potential limitation is that the outcomes are self‐rated psychological scales; therefore, the potential influence of positive expectations resulting from the intervention on the outcomes cannot be excluded.^35^ Self‐report outcomes may be susceptible to bias due to reliance on respondents' subjectivity and memory. The protocol mitigates this by employing outcomes comprising specific, behaviorally anchored questions (e.g., the GARS‐j and the LSAS‐j), which clearly define the timeframe and frequency of symptoms and aim to facilitate participants' accurate reporting of their actual symptoms. To minimize recall bias, links to the online questionnaires will be promptly emailed to participants at each assessment point. The online format of responses may also reduce socially desirable responses compared with in‐person assessments.^36^ This may encourage participants to report their actual clinical symptoms without hesitation.

This trial excludes participants with a formal diagnosis of SAD during screening. Consequently, the evidence presented will pertain to a non‐clinical population (young adults at high risk), which will limit its generalizability to clinical settings. The efficacy of those interventions for gaze anxiety in individuals already diagnosed with SAD should therefore be verified in a clinical setting.

This trial protocol has several strengths. Outcomes will be assessed not only post‐intervention but also at the 6‐month follow‐up, which should enhance the estimation of true effectiveness. The ITT principle allows for the derivation of unbiased conclusions regarding the effectiveness of the intervention.^37^ The sensitivity analyses could show the robustness of the results from the primary analysis.^38^ The statistical analysis plan will evaluate both continuous outcome data and clinically meaningful categorical variables (treatment response, remission, and worsening). Furthermore, the multisite design across numerous Japanese research institutions aims to achieve an adequate sample size and mitigate selection and facility biases.

This trial protocol was designed to investigate the efficacy of self‐help CBT programs on gaze and social anxiety in young adults. Despite some limitations, the multiple strengths could yield novel evidence.

## AUTHOR CONTRIBUTIONS

All authors contributed to the development of this trial protocol or the formulation of the statistical analysis plan, critically reviewed this manuscript, and approved the paper for submission.

## CONFLICT OF INTEREST STATEMENT

The first author (K.M.) is the author of the self‐help book used in one of the interventions. The other authors declare no conflicts of interest.

## ETHICS APPROVAL STATEMENT

This clinical trial protocol has been approved by the Institutional Review Board of Kagoshima University Hospital, through review (Approval Number: 240229). This clinical trial is registered in the University Hospital Medical Information Network Center, and an overview of the research protocol is publicly available (UMIN000057484).

## PATIENT CONSENT STATEMENT

N/A.

## CLINICAL TRIAL REGISTRATION

University Hospital Medical Information Network (UMIN) 000057484; https://center6.umin.ac.jp/cgi-open-bin/ctr/ctr_view.cgi?recptno=R000065681.

## Supporting information

Supporting Information.

## Data Availability

The data that support the findings of this study are available on request from the corresponding author. The data are not publicly available due to privacy or ethical restrictions.
