# Supplementary material for: Self‐help cognitive behavioral therapy for gaze anxiety in young adults: Protocol of a 3‐arms, multicenter, randomized controlled trial
Source: PCN Rep. 2025 Nov 4;4(4):e70236. doi: 10.1002/pcn5.70236 (PMC12583993; doi:10.1002/pcn5.70236)
Supplement: Supplementary file 1 — Supporting Information. [file PCN5-4-e70236-s001.pdf]

日本語版視線不安評価尺度 GARS-J（The Gaze Anxiety Rating Scale – Japanese Version）

GARS-J は、視線を合わせることに不安を感じる人の臨床症状や治療反応性を評価するために開発されました。GARS-J は 17 項目の行為状況または社会状況について、「恐怖感/不安感」と「回避」の程度を 0 ～ 3 までの 4 段階で評価、点数化し、総合得点から重症度を評価します。

日本語版視線不安評価尺度

お願い：**この 1 週間**にあなたが感じていた様子に最も当てはまる番号に、項目ごとに 1 つだけ選んで記入してください。項目をとばしたりせず、全て回答してください。

項目	恐怖感/不安感<br>0：全く感じない<br>1：少しは感じる<br>2：はっきりと感じる<br>3：非常に強く感じる	回避<br>0：まったく回避しない<br>1：少しだけ回避する<br>2：ある程度は回避する<br>3：たくさん回避する
1. スピーチする	0 1 2 3	0 1 2 3
2. パーティで大勢の人に向けて話す	0 1 2 3	0 1 2 3
3. 会議で発言する	0 1 2 3	0 1 2 3
4. 数人での議論中に話す	0 1 2 3	0 1 2 3
5. 買い物でレジ係とやり取りする	0 1 2 3	0 1 2 3
6. 自己紹介する	0 1 2 3	0 1 2 3
7. 偶然出会った知り合いに挨拶する	0 1 2 3	0 1 2 3
8. あまり知らない人に話しかける	0 1 2 3	0 1 2 3
9. 魅力的だと思う人に話しかける	0 1 2 3	0 1 2 3
10. まだあまり親しくない人を招待する	0 1 2 3	0 1 2 3
11. 愛する誰かと親しみを感じたとき	0 1 2 3	0 1 2 3
12. 上司や先生と自分の作品の質を話し合う	0 1 2 3	0 1 2 3
13. 親しい家族と決まりきった会話をする	0 1 2 3	0 1 2 3
14. 自分に向かって話す人の話をきく	0 1 2 3	0 1 2 3
15. 自分の話を聞いている人に向かって話す	0 1 2 3	0 1 2 3
16. 相手の意見に同意できないと伝える	0 1 2 3	0 1 2 3
17. ほめられる	0 1 2 3	0 1 2 3
**総　計**（範囲 0～102）		

日本語版視線不安評価尺度の質問項目は、鹿児島大学松本一記が翻訳しており、かつ福井大学濱谷沙世が逆翻訳した文章をオリジナル版の著者 Dr. Franklin R. Schneier が確認しています。文献. Schneier FR, Rodebaugh TL, Blanco C, Lewin H, Liebowitz MR. Fear and avoidance of eye contact in social anxiety disorder. Compr Psychiatry. 2011;52(1):81-87. Doi:10.1016/j.comppsych.2010.04.006.
